# Investigation of Fe-Ni Battery/Module for Grid Service Duty Cycles

**DOI:** 10.3390/ma17122935

**Published:** 2024-06-15

**Authors:** Nimat Shamim, Edwin C. Thomsen, Alasdair J. Crawford, Vilayanur V. Viswanathan, David M. Reed, Vincent L. Sprenkle, Guosheng Li

**Affiliations:** 1 Battery Materials & System Group, Pacific Northwest National Laboratory, Richland, WA 99352, USA; nimat.shamim@pnnl.gov (N.S.); edwin.thomsen@pnnl.gov (E.C.T.); vilayanur.viswanathan@pnnl.gov (V.V.V.); david.reed@pnnl.gov (D.M.R.); vincent.sprenkle@pnnl.gov (V.L.S.); 2 System Optimization Group, Pacific Northwest National Laboratory, Richland, WA 99352, USA; alasdair.crawford@pnnl.gov

**Keywords:** Fe-Ni battery, grid storage, duty cycle, frequency regulation

## Abstract

Iron–nickel (Fe-Ni) batteries are renowned for their durability and resilience against overcharging and operating temperatures. However, they encounter challenges in achieving widespread adoption for energy storage applications due to their low efficiency and the need for regular maintenance and electrolyte replacement, which adds to maintenance costs. This study evaluates and demonstrates the capabilities of Fe-Ni batteries for participating in grid energy storage applications. Stable performance was observed frequency regulation (FR) testing at 100% and 50% state of charge (SOC)s, while at 50% SOC, there was a 14% increase in efficiency compared to 100% SOC. Although 25% SOC achieved higher efficiency, limited cyclability was observed due to reaching the discharge cutoff voltage. Optimal SOC selection, battery monitoring, maintenance, and appropriate charging strategies of Fe-Ni batteries seem to be crucial for their FR applications. Fe-Ni batteries exhibit stable peak shaving (PS) results, indicating their suitability and reliability under various load conditions for PS testing. Extended cycling tests confirm their potential for long-term grid-scale energy storage, enhancing their appeal for PS and FR applications.

## 1. Introduction

Battery energy storage systems (BESS) have emerged as a key technology in modern grid applications due to their ability to address issues related to renewable energy integration, grid stability, energy arbitrage, and backup power. The application of BESS in the electricity grid is becoming more widespread as it has the potential to transform the electricity grid by making it more reliable and sustainable. While different types of battery chemistries are demonstrated for grid energy storage, some of the most popular battery energy storage technologies in use today include lithium-ion batteries (LIBs), lead acid batteries, flow batteries, and high-temperature sodium batteries [1,2,3,4]. However, due to various challenges, such as high cost, safety issues (fire hazard), limited cycle life, high-temperature operation, and a large footprint, associated with current battery technologies [5,6], enormous efforts are still required for battery development and validation to further improve BESS.

One promising battery technology for energy storage is the iron–nickel (Fe-Ni) battery, also known as the Edison battery, which was first invented by Thomas Edison in the early 1900s [7,8]. These batteries offer several advantages, such as durability, high-temperature tolerance, safety, a long cycle life and calendar life when used for various grid services [9,10,11]. Fe-Ni batteries can operate in extreme temperatures ranging from −40 °C to 60 °C [12]. This makes them suitable for deployment in a variety of environments, including hot and cold climates. In contrast to LIBs, which have a high cost and use scarce materials such as lithium and cobalt, the nickel and iron used as the main components in Fe-Ni batteries are abundant in the earth’s crust, and furthermore, Fe-Ni batteries are fully recyclable, making them a sustainable option that are less susceptible to material supply chain issues.

**Figure 1:**
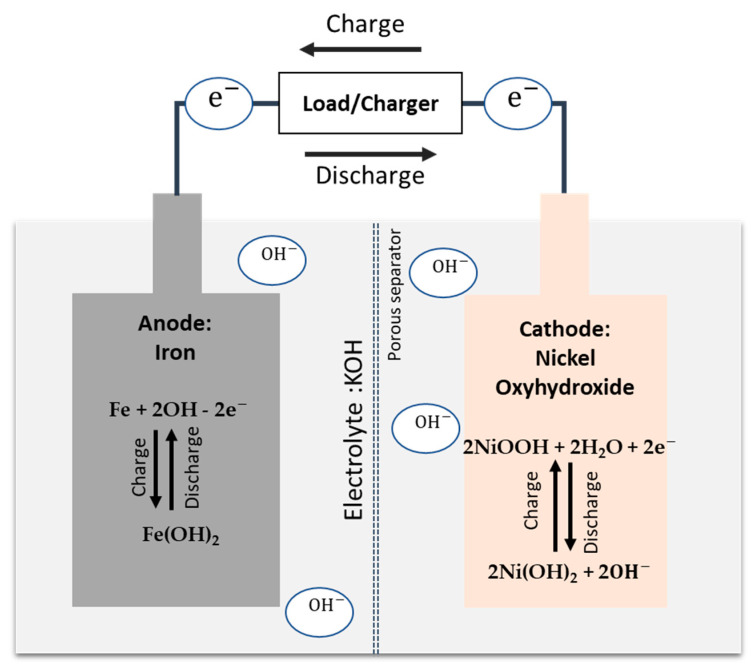
Schematic view of a Fe-Ni battery and its redox reactions at cathode and anode, respectively.

A typical Fe-Ni battery shown in Figure 1 consists of alkaline electrolytes (mainly a high-concentration potassium hydroxide aqueous solution, KOH), a nickel oxyhydroxide (NiOOH) cathode, and an Fe anode in the charged state [13]. During discharge, the Fe anode is oxidized to form iron hydroxide (Fe(OH)_2_), and the NiOOH in the cathode is reduced to form nickel hydroxide (Ni(OH)_2_). The reactions in the cathode and anode can be expressed as follows [7,14]:

(1) $$Anode:Fe+2{OH}^{-}-2e^{-}⇌Fe{\left(OH\right)}_{2}\;\;-0.88\;V\;\text{vs.}\;\mathrm{SHE}$$

(2) $$Cathode:2NiOOH+2H_{2}O+2e^{-}⇌2Ni{\left(OH\right)}_{2}+2{\left(OH\right)}^{-}\;\;0.52\;V\;\text{vs.}\;\mathrm{SHE}$$

(3) $$Overall:Fe+2NiOOH+2H_{2}O⇌Fe{\left(OH\right)}_{2}+2Ni{\left(OH\right)}_{2}\;\;E_{0}\;=\;1.4\;V$$

(4) $$HER:2H_{2}O⇌H_{2}+{2OH}^{-}\;\;-0.83\;V\;\text{vs.}\;\mathrm{SHE}\;(\mathrm{pH}∼14)$$

Typical Ni-based alkaline batteries, such as Fe-Ni, Ni-Cd, Ni-Zn, Ni-H_2_, and Ni-metal hydride batteries, feature a nickel oxyhydroxide (NiOOH) electrode as the charged cathode [15]. Despite the variations on the anode side, the reactions on the cathode side in Ni-based batteries remain the same as mentioned in Equation (2). For Fe-Ni batteries, the potential of hydrogen evolution is typically around −0.83 V vs. the standard hydrogen electrode (SHE), and for Fe/Fe(OH)_2_, the redox reaction potential is close to −0.88 V compared to SHE. As the Fe-Ni battery charges to high state-of-charge (SOC), gas evolution occurs and water in the electrolyte is depleted. Hence, the Fe-Ni battery requires regular watering to top up the electrolyte. It also should be noted that hydrogen evolution reaction (HER) leads to a decrease in the Coulombic Efficiency (C.E.) since a part of the electrons during the charge is consumed in the HER [16,17], where the C.E. is the ratio of discharge capacity to the preceding charge capacity. It should be noted that the self-discharge rate of Fe-Ni batteries has been extensively characterized in the past. A consensus has been established that these batteries exhibit a relatively high self-discharge rate, with a 15% capacity depletion within the initial 10-day period, which is notable when compared to other rechargeable battery technologies like LIB [18].

Controlling hydrogen evolution is crucial for the stable and reliable operation of Fe-Ni batteries. Exploring ways to suppress HER kinetics has been one of the important research topics for improving the overall performance of Fe-Ni batteries. The efforts to enhance anode performance are primarily concentrated on mitigating HER. This includes the development of advanced additives [19,20,21,22], which elevate the overpotential for HER [23,24] through innovative designs of Fe electrodes [25,26], or adding catalysts [23,24] to facilitate in-situ hydrogen–oxygen recombination in the Fe-Ni batteries, thereby increasing the longevity of the battery system and reducing the maintenance cost from regularly watering Fe-Ni batteries. Some studies have also introduced unconventional approaches, including the use of HER inhibitors that adsorb onto electrode surfaces, thereby reducing the active sites available for water electrolysis, as well as anticatalysts that bind with HER intermediates to facilitate the occupation of active sites by energy storage charge carriers [27,28]. Research focused on enhancing the performance of the Ni(OH)_2_ positive electrode primarily revolves around three main objectives: enhancing the intrinsic conductivity of the electrode [29,30,31,32,33,34,35], increasing the electrochemically active surface area [31,36,37,38], and improving the structural stability of the electrode [39,40,41,42,43]. 

Testing plays a crucial role in evaluating the performance and reliability of Fe-Ni batteries for various grid applications. In this paper, four key testing methods were conducted to assess the capabilities of Fe-Ni batteries. The reference performance test method evaluates overall battery performance under standardized conditions to establish baseline performance metrics. By assessing factors such as energy capacity, efficiency, and cycle life, reference performance testing helps determine the suitability of batteries for specific grid applications and provides a benchmark for comparison. Measuring the resistance within the battery provides insights into its internal health and degradation over time. Identifying abnormalities impacting performance and reliability allows for proactive maintenance and ensures optimal battery operation, minimizing the risk of unexpected failures. The battery is tested for grid applications such as PS and FR duty cycles to test its suitability for such applications. The table below summarizes the tests conducted on the Fe-Ni battery. We conducted tests on the single cells and modules consisting of a series of connected cells to gain insights into their performance. Our analysis included evaluating the charge–discharge scheme of the battery, C.E., energy efficiency, and degradation during PS and FR duty cycles. The findings in the paper provide valuable insights into the use of Fe-Ni battery technology for grid energy storage applications.

## 2. Experimental

### 2.1. Module Specification

**Figure 2:**
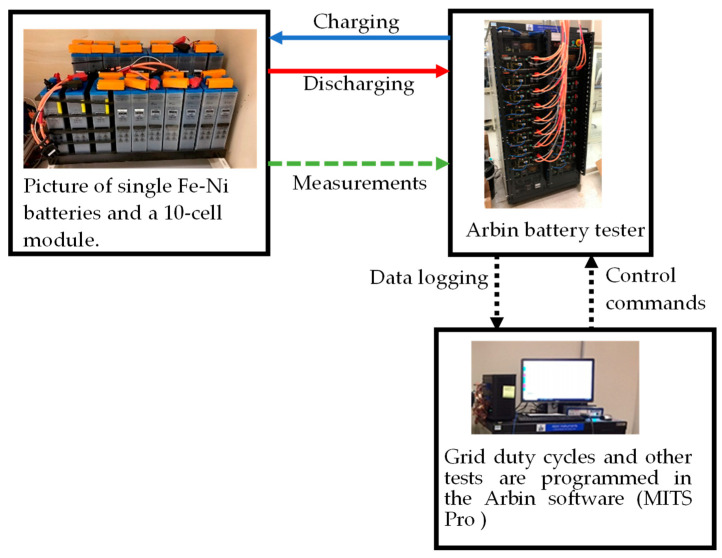
Experimental setup of the Fe-Ni battery/module test.

The battery module tested in this work is a Fe-Ni battery module, a commercial off-the-shelf production (Iron Edison, Denver, CO, USA), with a rated capacity of 100 Ah at the 5 hr rate. Figure 2 shows the experimental setup for the battery test, where the Fe-Ni battery is the DC storage block, and the Arbin battery cycler performs the role of the grid and site controller. The Arbin battery cycler charges and discharges the battery, capturing all necessary measurements. With a measurement precision of 100 ppm (0.01%), the Arbin equipment ensures highly accurate data collection. Table 1 presents the technical specifications of the Fe-Ni battery with 10 cells in series used for testing.

### 2.2. Capacity Test Protocols

A standard capacity test for Fe-Ni cell begins by bringing the battery to a full 100% SOC by applying a bulk charge at the C/4 rate (25 A for 100 Ah cell) until the voltage of the cell reaches 1.65 V. This is followed by an absorption charge, which consists of holding the cell voltage at 1.65 V for 4–5 h. Then, the battery is discharged at C/4 current rate (25 A) until reaching the discharge cutoff voltage at 1.0 V. The charge and discharge cycles are repeated twice to verify the capacity. The capacity is measured using Coulomb counting and the SOC is calculated as a percentage of the battery’s total capacity. The test protocol follows the document “Protocol for Uniformly Measuring and Expressing the Performance of Energy Storage Systems” [44]. 

According to the manufacturer’s operating manual, the minimum charge, the optimal charge, and the maximum charge rates are C/20 (5 A), C/4 (25 A) and C/2 (50 A), respectively. In addition to the standard capacity procedure described above, to understand various battery characteristics, capacity tests are performed at different conditions, such as different bulk voltages, different bulk charge rates, different absorption times (up to 5 h), different percent overcharge in absorption stage (up to 30% of previous cycle discharge capacity) and different discharge rates. 

Capacity cycling is also performed long term (>100 cycles) to observe cell degradation with time and cycles. The results of capacity tests are discussed in the Capacity Test Results section.

### 2.3. Frequency Regulation Testing 

The FR duty cycle is energy neutral, with each FR cycle lasting 24 h and consisting of many short, partial charge and discharge pulses that change every 4 s. This work uses an FR duty cycle published in the Unified Protocol for Measuring and Expressing Energy Storage System Performance developed by energy storage industry stakeholders and led by Pacific Northwest National Laboratory and Sandia National Laboratories [44]. FR tests are performed at three different initial state of charge (SOC) levels (100%, 50%, and 25%) to observe the stability, performance, and degradation effect on the battery cells. After each FR cycle, one 24 h power profile is considered as one cycle, and the battery or multi-cell string is recharged back to its original SOC before continuing to the next FR cycle.

### 2.4. Peak Shaving Test Protocols

A PS duty cycle test for a battery involves testing the battery’s ability to shave or reduce peak loads on an electrical system during periods of high demand. This is typically performed by charging the battery during periods of low demand, such as early morning, late afternoon, or early evening peak periods. These peaks can be 2 to 6 h long. The battery is charged during off-peak hours, typically at night or during periods of low demand. A single Fe-Ni cell and a string of 10 cells connected in series are subjected to PS duty cycles to monitor the battery performances under different load conditions and duration. Discharge is performed at three discharge energies of 100 Wh, 80 Wh, and 60 Wh, and three discharge durations of 2 h, 4 h, and 6 h. The discharge durations are selected to represent real-world peak demand periods [44]. The recharge scheme is kept the same for all the tests. After each PS discharge half-cycle, the battery is charged at a constant power of 40 W until the voltage reaches 1.65 V, followed by an absorption charge until a 30% overcharge of the previous discharge capacity is achieved. The 10-cell string is subjected to the PS duty cycle with a discharge energy of 1000 Wh for 6 h. The recharging of the 10-cell string follows the same procedure as the single-cell test with the bulk charge power at 400 W to 16.5 V, followed by the absorption phase. 

### 2.5. Resistance Measurement

An internal resistance test is performed using the integrated capabilities of the Arbin cycler (16-bit control and measurement resolution, and 0.05% FSR accuracy). The cycler employs a direct current (DC) pulse methodology involving 10 charge and discharge pulses. The voltage values are picked up at the end of the pulse current and at the start of the rising current. It calculates the internal resistance using Ohm’s law, which states that voltage is divided by current, and an average of 10 pulses is adapted to provide a more accurate representation. The pulse width can be varied from 1 millisecond (ms) to 1000 ms.

## 3. Results and Discussion

### 3.1. Capacity Test Results

**Figure 3:**
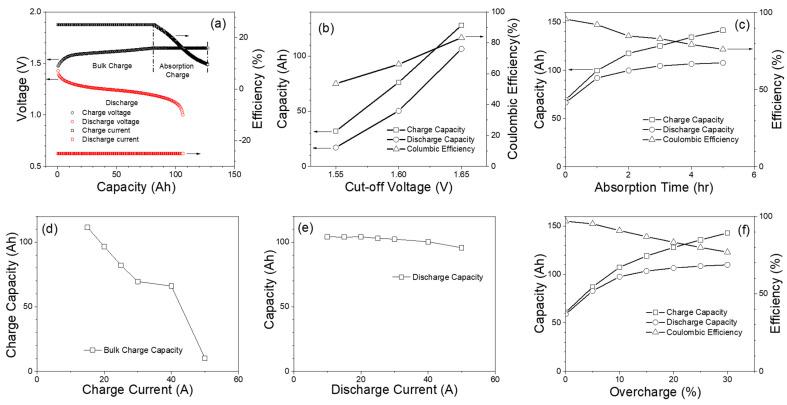
(**a**) Capacity vs. voltage and current profile (**b**) Charge cutoff voltage vs. capacity (**c**) capacity vs. absorption time (**d**) capacity at different charge current rate (**e**) capacity at different discharge current rate (**f**) capacity vs. overcharge (%).

The primary purpose of the capacity test is to determine the capacity of the battery and provide an assessment of the performance and health of a battery. Capacity tests are performed under different conditions, including bulk cutoff voltage, absorption time, charge current, and overcharge percentage. Figure 3a shows typical voltage and current plots vs. the capacity of the Fe-Ni battery tested in this work. During bulk charging at a constant current of 25 A, the voltage of the battery increases gradually until it reaches the bulk cutoff voltage of 1.65 V per cell. During the following absorption charge step, the battery is charged at the constant voltage mode, in which the voltage remains at a specific level (e.g., 1.65 V) per cell for a certain amount of time, such as 5 h. During this step, the charge current gradually decreases as the battery state of charge (SOC) increases. This two-step charge process allows the battery to be fully charged to its 100% SOC. At the beginning of the constant current discharge, the battery voltage starts at around 1.4 V per cell and decreases gradually throughout the discharge. As the Fe-Ni battery approaches the end of available capacity, the voltage starts to drop rapidly, so the cutoff discharge voltage is set at 1.0 V to avoid any adverse reactions, such as the formation of the less reversible iron oxyhydroxide (FeOOH) phase at lower discharge voltages [45,46]. The charge and discharge cycles are repeated for 10 cycles to validate their stable performances.

Figure 3b shows how the battery charge capacity changes when the bulk cutoff voltage increases from 1.55 V to 1.65 V while keeping the charge (bulk charging)/discharge current and the absorption time at 25 A and 5 h, respectively. It is worth mentioning that charge/discharge capacity and C.E. increase as the bulk cutoff voltage increases from 1.55 to 1.65 V, which indicates that the bulk cutoff voltage plays an important role in suppressing the gas evolution kinetics mentioned in Equation (4) towards the higher SOCs of Fe-Ni battery. C.E. for the capacity test is measured using the following method.

(5) $$Coulombic\;efficiency\;(C.E.)=\frac{Discharge\;capacity}{Charge\;capacity}$$

Absorption time is also varied from 0 up to 5 h to observe the capacity and efficiency of the Fe-Ni battery, and Figure 3c shows the plots of capacity and C.E. vs. absorption time at bulk charge/discharge current of 25 A and bulk charge voltage cutoff of 1.65 V. While longer absorption time increases the charge capacity of the battery, the efficiency decreases with increasing absorption time. This is because during the absorption period, part of the charging current promotes gas evolution due to the generation of hydrogen and oxygen by side reactions, resulting in a decrease in Coulombic efficiency. Figure 3d shows that the charge capacity of the bulk charge decreases as the charge rate is increased from C/6.6 (15 A) to C/2 (50 A) as the bulk charge voltage cutoff is fixed at 1.65 V. Similarly, Figure 3e shows that when the discharge current increases, the discharge capacity decrease slightly, which is likely due to the larger voltage drops from the higher currents. 

As shown in Figure 3c, the absorption time is an important factor for achieving a high capacity of Fe-Ni batteries. Instead of ending the absorption charge after a fixed amount of time, as shown in Figure 3c, an overcharge relative to the discharge capacity of the previous cycle is given as an end-of-charge condition. Figure 3f shows that with an increasing overcharge percentage, the charge and discharge capacity increase, and C.E. decreases as expected. Maintaining an optimal overcharge percentage relative to the discharge capacity is expected to improve battery performance in terms of high discharge capacity at reasonably high C.E. without excessive gas evolution. The overall efficiency of the battery shows a similar value that has been reported in the literature for large-size Fe/Ni cells [7].

**Figure 4:**
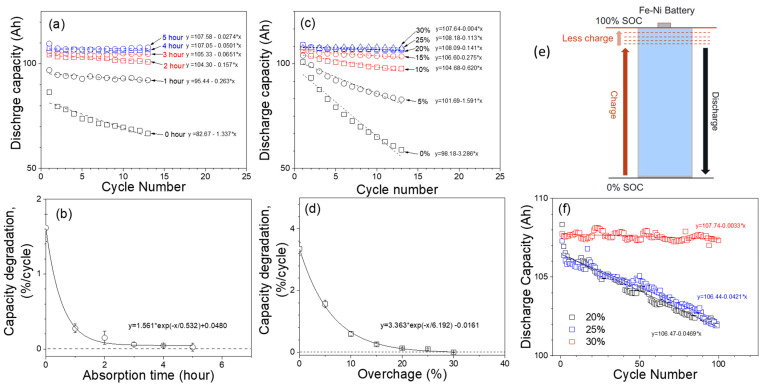
(**a**) Discharge capacity plots for different absorption times (0–5 h). (**b**) Capacity degradation rate (%/cycle) for different absorption times. (**c**) Discharge capacity plots for different overcharges (0–30%). (**d**) Capacity degradation rate (%/cycle) for different overcharges. (**e**) Capacity degradation mechanism due to deficient charge. (**f**) Long-term cycle for Fe-Ni batteries with overcharges of 20, 25, and 30%, respectively.

For long-term cycling, it is important to have an optimized absorption time or overcharge (%), which ensures less reversible capacity loss per cycle. Figure 4a shows the discharge capacity of thirteen cycles at absorption time, varying from 0 h to 5 h. By linearly fitting the capacity decay shown in Figure 4a, it is possible to estimate the % reduction in capacity per cycle for different absorption times (Figure 4b). As the absorption time is increased, reversible capacity loss per cycle is reduced. At an absorption time of three, four, and five hours, the capacity loss per cycle is close to zero, while the percent loss per cycle increases exponentially at an absorption time of less than two hours. Similarly, Figure 4c shows the discharge capacity of thirteen cycles at overcharge, varying from 0 percent to 30% based on the discharge capacity from the previous cycle. Figure 4d shows the percent capacity degradation per cycle (calculated as the slope from Figure 4c divided by initial capacity) vs. overcharge percentage. One common observation for the tests with different absorption times and percent overcharges is that the capacity loss is much lower for long absorption times or large overcharges. It is worth noting that the observed capacity loss is not permanent, as the full capacity can be restored by deeply charging/discharging the batteries for a few cycles. Therefore, one postulates that the capacity loss at short absorption time/low overcharge is caused by insufficient charging of the battery. As shown in Figure 4e, the battery SOC at the end of the charge decreases with each cycle. Hence, after each cycle, the battery loses some of its capacity. By controlling the absorption time or overcharge percentage during the charge, one can maintain a stable battery capacity for long-term cycling. Figure 4f shows the capacity trend of Fe-Ni cells tested up to 100 cycles at 20%, 25% and 30% overcharge, respectively. For 20% and 25% percent overcharges, the battery loses capacity at a rate of 0.04%/cycle, resulting in a loss of 4.2 Ah after 100 cycles. However, the capacity loss rate can be significantly reduced to 0.003%/cycle (0.3% capacity loss every 100 cycles) for a 30% overcharge. 

The battery operating SOC range determines the charge or energy capacity, while the charge/discharge rate, percent overcharge and operating SOC range determine the C.E. and round-trip efficiency (RTE). As noted earlier, the available charge or energy capacity to a fixed end of discharge condition increases with an increasing percent overcharge, while the Coulombic and round-trip efficiency decrease, with a corresponding decrease in operation and maintenance (O&M) costs related to increased gas evolution. For storage used in various grid services, increasing the discharge energy throughput while maintaining a high RTE, with corresponding lower electricity costs for charging and lower O&M costs, is key to achieving a low levelized cost of storage (LCOS), which is a key metric to compare the suitability of various storage technologies for grid services. Our approach allows the choice of suitable charge–discharge rates and the selection of an optimal percent overcharge to allow operation across an SOC range that allows high available energy capacity at the end of charge, high RTE and low O&M.

Determining the SOC of a Fe-Ni battery is not straightforward. A common method of determining the SOC of a battery is by using Coulomb counting. Coulomb counting involves measuring the amount of charge flowing into or out of a battery by integrating the current over time. For Fe-Ni batteries, coulombs corresponding to the desired reactions (Equations (1)–(3)) are likely to be less than the total coulombs during the charge due to parasitic reactions such as HER, and thus cannot provide accurate results.

**Figure 5:**
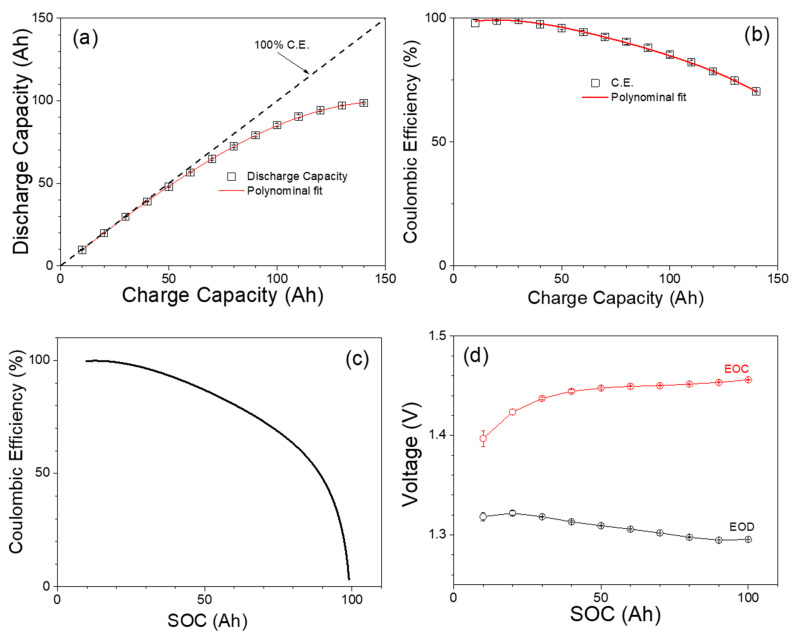
(**a**) Discharge capacity of Fe-Ni battery vs. charge capacity. (**b**) Coulombic efficiency of Fe-Ni vs. charge capacity. (**c**) Coulombic efficiency vs. SOC. (**d**) End of charge (EOC) and End of discharge (EOD) voltage vs. SOC.

Another method for SOC measurement is to monitor the battery open circuit voltage (OCV) and compare it to an OCV vs. SOC curve. Three battery cells are tested to determine the OCV vs. SOC curve to verify the result consistency. Each battery cell is charged from a fully discharged state at various charge capacities and discharged at a current of 25 A until the voltage reaches the discharge cutoff voltage of 1.0 V. Figure 5a shows the discharge capacity of the battery cells as a function of charge capacity. The relationship shows a nonlinear trend wherein an increase in charge capacity does not result in a proportional increase in discharge capacity. Notably, at lower charge capacities, the slope of discharge capacity versus charge capacity is 1, corresponding to a C.E. of 100%. However, with increasing charge capacity, the battery’s capacity to deliver an equal discharge capacity diminishes, leading to a departure from the initial 45-degree linear region and a reduction in C.E. This nonlinear behavior can be attributed to the gas evolution reaction during charging. Figure 5b shows the C.E. vs. charge capacity curve. The curve shows that as the charge capacity increases, the C.E. decreases. 

Continuing this analysis, in Figure 5c, the C.E. is plotted against the SOC of the battery cells. The SOC in this context is the discharge capacity value corresponding to the charge capacity value in Figure 5b. To calculate the C.E. at each SOC level, the derivative of the discharge capacity curve from Figure 5a is taken. This derivative represents the rate of change of discharge capacity with respect to charge capacity and helps quantify the C.E. at different levels of battery SOC. At lower SOC values, the C.E. tends to be higher, close to 100%, reflecting more efficient charge and discharge processes. However, as the SOC increases, the C.E. decreases at a slower rate up to SOC of 70 Ah, followed by a much steeper decrease at SOC > 80 Ah, with the C.E. dropping from 70% at 70 Ah to 3% at 98 Ah, signifying the potential losses associated with higher charge levels.

Figure 5d presents the correlation between SOC and the end-of-charge (EOC) as well as end-of-discharge (EOD) voltages. The EOC and EOD voltages are measured subsequent to a one-hour period of rest. This graph representation holds considerable significance due to its potential to enable the estimation of the battery state of charge through voltage measurements. By analyzing the voltage values at the end-of-charge and discharge cycles, an approximate determination of the battery’s SOC becomes feasible. This graph serves as a pivotal tool in subsequent battery cycling tests, offering valuable insights on battery SOC to make informed operational decisions.

### 3.2. Frequency Regulation Test Result

#### 3.2.1. Frequency Regulation at 100% SOC

FR cycling is performed at an initial SOC of 100% for Fe-Ni batteries. During FR cycling, the battery is expected to absorb excess power from the grid during periods of high generation. Hence engaging in FR at 100% SOC is infrequent due to the limitations posed by restricted charge acceptance, higher degradation rate and safety concerns related to overcharging, especially for lithium-ion batteries [47,48]. However, Fe-Ni batteries can handle overcharging, and hence are ideal for absorbing power during periods of over-generation, and providing power during periods of under-generation, ensuring grid stability and reliability. 

While the FR signal is energy neutral, the SOC is expected to drift lower during this FR duty cycle. However, operating the battery at close to 100% SOC allows the battery to participate in the FR market, while ensuring that the battery has maximum stored energy available to respond to emergencies caused by unexpected power shortages.

**Figure 6:**
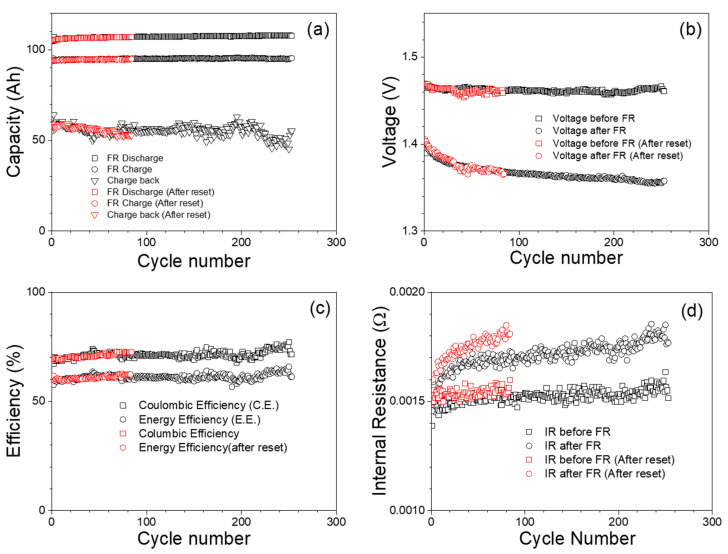
FR at 100% SOC. (**a**) Charge, discharge, and charge-back capacity. (**b**) Voltage plots for before and after FR at 100% SOC. (**c**) Coulombic and energy efficiency of FR at 100% SOC. (**d**) Internal resistance of the battery before and after each FR cycle.

Figure 6 presents the outcomes of FR cycling performed at the SOC of 100%. In Figure 6a, the graph displays the charge and discharge capacities witnessed during cycles of FR, along with the recharge capacity. Following each FR cycle, the battery is charged back to 100% SOC. Notably, the charge and discharge capacities observed during the FR cycles remain consistent across all cycles. Moreover, the stability observed in the charge-back capacity implies the battery’s capability to maintain its target end-of-charge SOC level.

Figure 6b illustrates the voltage before and after the FR duty cycle, where the FR duty cycle consists of a 24 h signal without the charge-back step. The voltage, measured after a 1 h resting period, exhibits stability prior to each FR cycle, affirming the procedure’s ability to maintain the battery SOC at 100% after the charge-back step. The voltage observed after the duty cycle declines slightly with each cycle. This decrease in voltage could potentially be attributed to cell degradation during the cycling process.

Figure 6c presents the Coulombic and energy efficiencies. The Coulombic and energy efficiencies are calculated as

(6) $$Coulombic\;efficiency=\frac{\sum {Discharge\;Capacity}_{FR}}{\sum {Charge\;Capacity}_{FR}+\sum Recharge\;Capacity}$$

(7) $$Energy\;efficiency=\frac{\sum {Discharge\;Energy}_{FR}}{\sum {Charge\;Energy}_{FR}+\sum Recharge\;Energy}$$

It is worth mentioning that the C.E. and E.E. presented in Equations (6) and (7) calculate the discharge and charge capacity or energy separately for the FR process, which alters its polarity every 4 s to follow the frequency changes of the grid. The observed C.E. and energy efficiency (E.E.) are around 70% and 55–60%, respectively. 

Figure 6d portrays the internal resistance before and after the FR duty cycle. Internal resistance measurement is performed with the ARBIN cyclers DC pulse test method at a 20 ms pulse width. Notably, the internal resistance, as shown in Figure 6d, exhibits a gradual increase as cycling progresses, possibly due to cell degradation during cycling. 

After completing 250 cycles, the battery underwent a resetting process, which involves fully discharging and charging the battery to restore it to its original state, if possible. In Figure 6, the data points represented in red color signify the battery’s behavior after this resetting process. A noteworthy observation is evident across Figure 6a–d: following the reset, the battery effectively returns to its initial operational state. In Figure 6a, the red data points indicate that after the reset, the battery’s capacity-related behaviors revert to their original values. Moving to Figure 6b, which showcases voltage behaviors before and after the FR duty cycle, the red data points once again signify the restoration of the battery’s voltage characteristics to their initial state following the reset. In Figure 6c, the Coulombic and energy efficiencies are depicted. The red data points suggest that post–reset, the battery’s efficiency behaviors align with their original levels. Lastly, Figure 6d illustrates the internal resistance before and after the FR duty cycle. The red data points confirm that subsequent to the reset, the battery’s internal resistance reduces and recovers to its original state. However, the rate of increase in internal resistance per cycle is slightly higher after resetting. This consistent pattern across all four graphs underscores the efficacy of the resetting process in bringing the battery’s behavior back to its original condition. The ability to reset the battery indicates its resilience and the potential to rejuvenate its performance characteristics, supporting prolonged operational life and efficient usage.

Overall, the Fe-Ni battery demonstrates its potential to perform FR applications at 100% SOC. To perform FR at an initial 100% SOC prior to each cycle, it is essential to monitor and maintain the battery properly and adjust the charging strategy to ensure optimal battery health.

#### 3.2.2. Frequency Regulation at Initial 50% SOC

**Figure 7:**
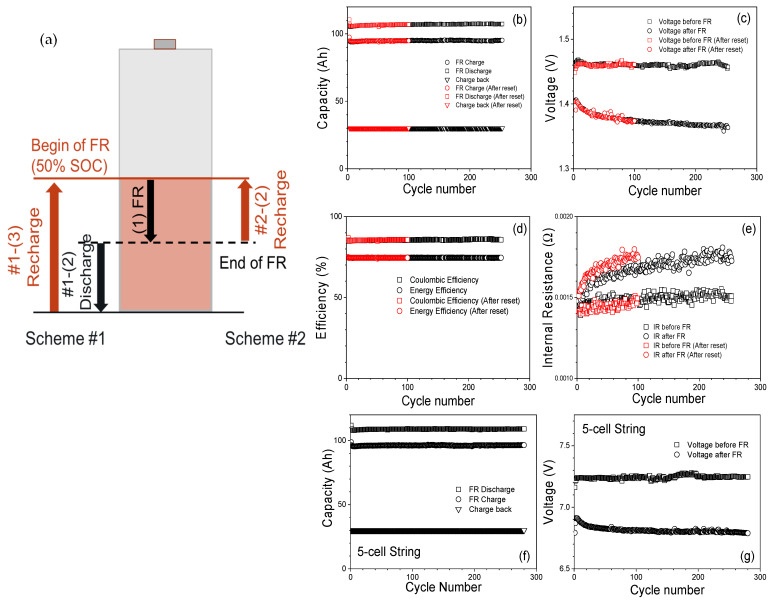
(**a**) Schematic diagram of two different FR protocols. (**b**) FR charge, discharge, and recharge back capacity for FR at 50% SOC. (**c**) Voltage plots for before and after FR at 50% SOC. (**d**) Coulombic and energy efficiencies. (**e**) Internal resistance of the battery before and after each FR cycle. (**f**) Charge, discharge, and charge-back capacities of FR at 50% SOC for 5-cell string. (**g**) Voltage plots for before and after FR at 50% SOC for 5-cell string.

For the FR test performed at an initial 50% SOC, the battery is discharged to 50% SOC from a fully charged state, and the same FR duty cycle is applied. After one FR cycle, the battery needs to be charged back to 50% SOC. Charging the battery back accurately to the target 50% SOC is difficult because Fe-Ni does not have a C.E. of 100%. The test is performed using two schemes. In scheme #1, shown in Figure 7a, step (1) is to apply the FR cycle. Step #1-(2) in Figure 7a is to fully discharge the battery, and step #1-(3) is to recharge the battery back to 50% SOC. This scheme is performed to understand the Coulomb count of the battery. Scheme #1 requires an additional discharge step, which reduces the availability of the battery for the FR service. In scheme #2, the battery is recharged back to 50% SOC without fully discharging the battery. The recharge capacity of step #2-(2) is adjusted based on the voltage vs. SOC curve shown in Figure 5d. Figure 7b shows the total charge capacity and discharge capacity during the FR cycle under scheme #2 and the bulk charge capacity during recharge. The results show bulk recharge capacity is stable after an FR cycle, which indicates that the battery’s SOC range during the FR cycle is maintained. Maintaining the same battery SOC range throughout FR cycling ensures that the battery performance and available energy capacity throughout FR cycling are predictable, thus improving the reliability of the provided grid service, that it remains efficient and that it can handle cycling. Figure 7c shows the voltage before the FR cycle and after the FR cycle. Each voltage is measured after one hour of rest. The voltages are stable, and, after reset, the voltages go back to the original values. 

Figure 7d shows that C.E. and energy efficiency both are very stable. It indicates that the battery is efficiently managing the charging and discharging processes and is effectively responding to the fluctuating power demand from the grid. The Coulombic and energy efficiency are greater than the values for FR performed at an initial SOC of 100%. Figure 7e shows the internal resistance before and after the FR cycle. It can be observed that the internal resistance increases with FR cycling at initial SOC of 50% in Fe-Ni batteries as observed in 100% SOC. 

Furthermore, the investigation extends to the application of FR at a 50% SOC using a 5-cell string configuration, The outcomes depicted in Figure 7f underscore the noteworthy stability of the capacity during FR charge, discharge, and charge-back processes, mirroring the consistent performance observed in individual cell testing. Additionally, Figure 7g illustrates the voltage profiles pre- and post-FR cycles, revealing remarkable stability in the voltage levels. These results collectively demonstrate that the deployment of a 5-cell string configuration effectively facilitates stable FR performance at 50% SOC. 

Conducting FR at an initial SOC of 50% for Fe-Ni batteries offers notable advantages. It allows these batteries to seamlessly provide FR services by either absorbing or injecting power, effectively aligning power supply with grid demand, and thereby contributing to grid stability. Operating Fe-Ni batteries around 50% SOC typically yields superior performance in terms of both energy efficiency and power delivery than operating around 100% SOC. This enhanced performance translates into more effective FR services, bolstering overall grid stability. The durability, extended cycle life, and robustness of Fe-Ni batteries make them exceptionally well-suited for the demanding requirements of FR applications.

#### 3.2.3. Frequency Regulation at 25% SOC

**Figure 8:**
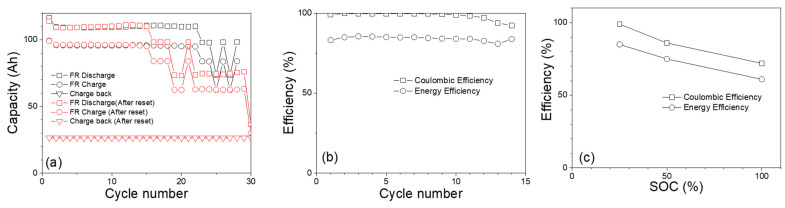
(**a**) Charge, discharge, and charge-back capacities for FR at 25% SOC. (**b**) Coulombic and energy efficiencies for FR at 25% SOC. (**c**) Comparison of Coulombic and energy efficiencies of FR duty cycles at 25, 50, and 100% SOCs.

Since the FR duty cycle operates within a limited range of SOC or capacity (~12 Ah) as shown in Figure 6a and Figure 8a, the FR cycle was further tested at lower SOC levels (e.g., 25%), which provides an opportunity to investigate whether obtaining a high C.E. by operating the Fe-Ni cell at a lower SOC, as shown in Figure 5c, is beneficial for FR testing. After each FR cycle, the battery was discharged until the voltage reached 1 V (~0% SOC) and then recharged to 25% SOC (Scheme #1 of Figure 7a). As shown in Figure 8a, after several consecutive FR cycles (up to 20 cycles), the capacity of the FR cycle could not maintain the expected value, which indicated that the FR cycle ended incompletely. It is found that the incomplete termination of FR cycles is caused by reaching the discharge voltage cutoff (1 V) during FR cycling. Fe-Ni batteries are known to exhibit voltage depression when they undergo extensive charge and discharge at lower SOCs. Over time, the voltage depression increases, causing the battery voltage to drop further and eventually hit the lower voltage cutoff. This degradation does not appear to be permanent, as resetting the battery that ended with an incomplete FR cycle by running a few full charge/discharge cycles appeared to be able to perform a limited FR cycle (about 15 cycles) again. 

Figure 8b shows the Coulombic and energy efficiencies of FR at 25% SOC is about 99% and 85%, respectively. Figure 8c compares the typical Coulombic and energy efficiencies for FR at 25, 50, and 100% SOCs. It is clear that both efficiencies are higher for lower SOCs; however, operating a Fe-Ni battery at 25% state of charge (SOC) for FR is not recommended due to the following reasons: At 25% SOC, the Fe-Ni battery has already discharged 75% of its energy capacity. In FR applications, where the battery needs to respond rapidly to grid fluctuations, the limited remaining capacity could lead to reduced performance, and the battery may not be able to provide the necessary power output when needed. The voltage of a battery is influenced by its SOC. Operating a Fe-Ni battery at a low SOC (such as 25%) increases the risk of voltage instability. As the battery continues to discharge during FR, its voltage will drop, potentially reaching the lower voltage limit, which could negatively affect the battery’s ability to deliver power to the grid effectively. To achieve better performance and longevity in FR applications, it is recommended to operate Fe-Ni batteries within an optimal SOC range (higher SOC than 25%) that will balance response time, capacity, and voltage stability. Table 2 shows a comparative analysis of FR duty cycles starting at a different initial SOC.

### 3.3. Peak Shaving Duty Cycle Test Results

**Figure 9:**
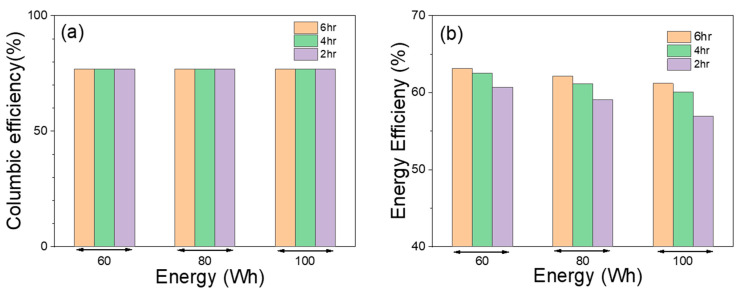
(**a**) Coulombic efficiency and (**b**) energy efficiency for PS with 100 Wh discharge energy at 2, 4, and 6 h duration.

A PS duty cycle test for a battery is an important step in evaluating the performance and suitability of a battery for use in PS applications and can help to ensure the battery can perform reliably and efficiently in real-world conditions. Single cells have been tested at various test scenarios; at three different discharge energy and discharge duration. The Table 3 shows the battery’s discharge energy (measured in watt-hours, Wh), discharge duration (h), discharge power (kW), discharge current (A), discharge capacity (Ah) and charge duration of the PS tests. Figure 9 shows (a) Coulombic efficiency and (b) energy efficiency of the same PS tests. Coulombic efficiency and energy efficiency for peak shaving duty cycle is calculated as

(8) $$Coulombic\;efficiency\;(C.E.)=\frac{Charge\;capacity}{Charge\;Capacity}$$

(9) $$Energy\;efficiency\;(C.E.)=\frac{Discharge\;Energy}{Charge\;Energy}$$

The Coulombic efficiency for all the discharge energy and discharge duration is about 76% because the battery is charged 30% above the discharge capacity in all the cases and the energy efficiency decreases as discharge duration decreases for the same discharge energy. In other words, when the battery is discharged at a higher power (shorter duration), it becomes less efficient at converting stored energy into usable energy. At high discharge currents, overpotential increases and causes it to lose more energy as heat rather than being delivered to the load. 

#### Long-Term Peak Shaving Duty Cycle

**Figure 10:**
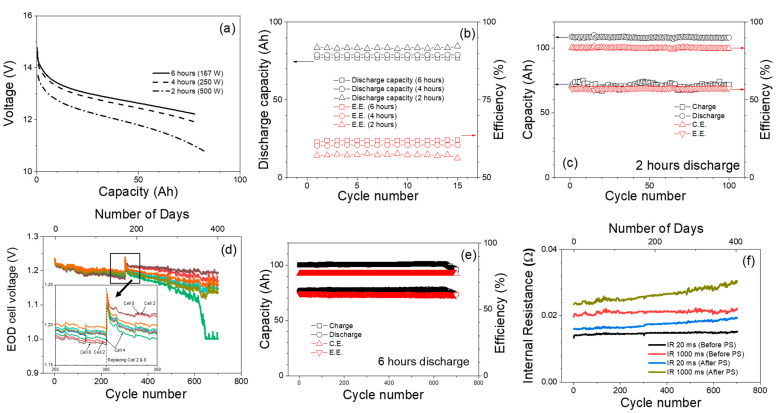
(**a**) Discharge voltage profiles for PS at 2, 4, and 6 h duration with a total discharge energy of (1000 Wh), respectively. (**b**) Discharge capacities for PS with 2, 4, and 6 h duration. (**c**) Capacity for charge and discharge at 2 h duration. (**d**) End of discharge voltage for each individual cell of the 10-cell string, different colors represents each battery cell (**e**) Charge/discharge capacity, Coulombic/energy efficiency of PS at 6 h duration for 10 cell string. (**f**) Internal resistance including ohmic (20 ms) and over polarization (1000 ms) for 10-cell sting over 700 PS cycles.

Long-term PS duty cycle testing on batteries is critical for evaluating performance, assessing reliability, estimating battery life, and ensuring safety. For the long-term testing, 10 cells are connected in series and subjected to a PS duty cycle, which has a discharge energy of 1000 Wh (100 Wh for each cell, 80% depth of discharge) discharge energy and a discharge duration of 6 h, 4 h and 3 h. Figure 10a shows the voltage vs. capacity plots at different discharge powers. At a discharge power of 250 W (4 h), the voltage curve exhibits slightly steeper declines compared to 167 W (6 h) discharge power, and discharging the battery at 500 W leads to a more pronounced voltage drop. The small increase in capacity at high discharge power is a result of decreased output voltage, which is due to high overpotential at high discharge powers. Figure 10b shows the discharge capacity and energy efficiency at different discharge durations/discharge power, energy efficiency decreases as discharge power increases. Figure 10c shows the test result for 2 h discharge up to 100 cycles. The charge/discharge capacity, and Coulombic (82%)/energy efficiency (56%) remain stable over 100 cycles. Figure 10d–f show the results of long-term testing performed at 6 h discharge duration for more than 700 cycles. Same as before, the total discharge energy is kept at 1000 Wh, and PS discharge power is 167 W, respectively. Figure 10d shows the end of discharge (EOD) voltage of individual battery cell after each PS cycle, with different colors representing each battery cell. It can be seen single cell EOD voltage kept decreasing over the cycles; particularly, cells two and eight showed the lowest EOD voltages among the 10 cells tested. At the 300th cycle, the PS duty test was paused, at the same time, cells two and eight were swapped with two new cells. Then, full charge and discharge cycles were performed before starting the PS duty cycle again. As shown in the Zoom-in of Figure 10d, the EOD voltages of cells two and eight were restored to the original value since they are new cells and outperformed other cells. Over time, the EOD voltage of these batteries continued to decrease over cycles, which indicates degradation of the battery performance, which could be caused by various causes, such as electrolyte deuteriation, electrode morphological change of electrode, etc. Around the 600th cycle, cell four started degrading more than the other cells and around the 650th cycle it reached the lower voltage limit (1 V) during the PS duty cycle. Figure 10e shows the trend of the charge/discharge capacity, Coulombic and energy efficiency of the 10-cell string over the long-term cycling. One can find that the discharge capacity and total charge capacity are mostly flat over the cycles until they reach around 650 cycles, which agrees with the trend shown in Figure 10d. It is also worth mentioning that the C.E. is about 77% throughout this long-term PS duty cycle, and this is due to the battery always being 30% overcharged compared to the discharge capacity. 

Figure 10f shows the internal resistance trends that were obtained from the pulse resistance measurements (20 ms and 1000 ms pulses, see experimental section for details) performed before and after each PS duty cycle. It should be noted that the resistance obtained from the short pulse (e.g., 20 ms) is more representative of the ohmic resistance; on the other hand, the resistance from 1000 ms pulse represents the overall polarization, including ohmic, charge transport and mass transport resistance. In general, the resistance measured before PS (100% SOC) is less than the resistance measured after PS (lower SOC), whether it is short or long pulses. This is mainly due to the formation of Fe(OH)_2_ as the battery discharges, and Fe(OH)_2_ is an electrically insulating material, which drastically interrupts the electron pathway on the anode. Another interesting observation is that the resistances obtained before PS are quite stable over the long-term cycling for either short or long pulses. In contrast, the resistance (short/long pulses) continued to increase after PS. A plausible reason for this is the fact that the EODV decreases with cycling as shown in Figure 10d, resulting in the battery SOC decreasing at the end of each PS discharge cycle. As noted earlier, as the SOC decreases, the resistance increases. 

Overall the testing results of the module consisting of 10 cells in serial connection and subjected to a PS duty cycle, which has a discharge energy of 1000 Wh.

From a materials supply chain standpoint, the Fe-Ni battery also presents advantages due to using readily available Fe, the most abundant among other common raw materials, and Ni, a popular raw material. It is worth mentioning that economic analysis of battery technologies for grid energy storage may not be that simple by just considering the raw materials cost, and should involve evaluating initial costs, lifespan, maintenance, efficiency, degradation rates, operational expenses, and other factors. For instance, Fe-Ni batteries have higher costs than lead–acid batteries but generally offer longer lifespans [49]. Beyond the factors mentioned, environmental and safety considerations, regulatory compliance, material availability, and integration costs with grid infrastructure are also crucial in determining the overall financial viability and cost-effectiveness of these systems. Nevertheless, Fe-Ni batteries are generally made of benign materials that do not pose substantial health and environmental concerns. The primary exception is the corrosive alkaline electrolyte, which can cause dermal burns if leakage occurs. However, this can be neutralized quickly and does not present long-term environmental hazards. Additionally, the nickel–iron battery construction allows for easy reprocessing, reuse, and revitalization of its components, contributing to its environmentally friendly profile [50]. Combined with the results presented in this work, Ni-Fe batteries could be a highly suitable option for grid applications, particularly where long-term sustainability and minimal environmental impact are priorities.

## 4. Conclusions

In conclusion, the capacity testing of Fe-Ni batteries, including varying conditions such as bulk voltages, absorption time, charge current rates, discharge current rates, and percent overcharge, has provided valuable insights into the battery’s characteristics and performance. The long-term capacity cycling tests have shown that Fe-Ni batteries can maintain their performance over an extended period.

The FR testing illustrated the potential of Fe-Ni batteries to participate in FR services, maintaining the stability of an electrical grid’s frequency through rapid charge and discharge cycles. Fe-Ni batteries show mixed results in FR applications at different SOCs since the battery’s efficiency and capacity vary depending on the SOCs. At 100% SOC, the battery shows stable performance for the tested FR duty cycle. The overall charge/discharge capacity and efficiency showed a stable trend for FR tests up to 250 cycles (250 days). Some observations on the voltage and resistance measured after each FR duty cycle indicated that degradation may occur during the FR duty cycle; however, it was successfully demonstrated that the performance of the Fe-Ni battery can be restored by exercising the battery to a couple of full charge/discharge cycles. At 50% SOC, the cell exhibits similar stable performance, but with better overall efficiency, about 14% higher than the FR duty cycle at 100% SOC. Although the FR duty cycle at 25% SOC achieves higher efficiency (13% higher than that at 50% SOC), the FR duty cycle at 25% SOC showed limited performance/cyclability (~20 cycles) due to reaching the discharge cutoff voltage during the FR duty cycle. Therefore, we concluded that it is crucial to choose the optimal SOC for specific applications, monitor and maintain the battery properly and implement appropriate charging strategies to ensure optimal performance, efficiency, and lifespan in FR applications.

The performance of Fe-Ni batteries during PS duty cycles has been demonstrated to be stable and reliable based on the conducted tests. The PS duty cycle test demonstrated the battery’s adaptability to different load conditions, discharging rates, and duration of discharge cycles. The long-term testing showcased the battery’s potential to perform well over several hundred cycles, further enhancing its attractiveness for PS applications.

Overall, this work shows that Fe-Ni batteries are a promising option for PS and FR applications. While the RTE of this chemistry is lower than competing technologies, this work has provided pathways to operate the Fe-Ni battery in an SOC range and C rates that mitigate side reactions, thus providing pathways to reduce operating and maintenance costs, while maintaining a stable Coulombic and energy capacity during long-term cycling.

## Figures and Tables

**Table 1:** Technical Specification of 10-cell stack Fe-Ni battery.

Nominal Voltage, V	12 V (1.2 V/cell)
Energy, kWh	1.2 kWh, 5 h rate
Cell Capacity, Ah	100 Ah, 5 h rate
Weight (filled), kg	6.4/cell
Electrolyte Vol. liter	1.5/cell
Dimension, cm	8.1 (w) × 14.2 (d) × 36.8 (h)/cell
Operating Temperature, °C	−30 °C To + 60 °C

**Table 2:** Fe-Ni battery performance of FR duty cycles.

Initial SOC for Starting FR	Charge/Discharge Capability	Energy Efficiency	Coulombic Efficiency	Stability
100%	More discharging capability	50–60%	70%	Stable
50%	Has room for both charge and discharge	70–75%	85–90%	Stable
25%	More charging capability	85%	99%	Unstable

**Table 3:** Experimental parameters and measured parameters for various PS tests.

Discharge Energy (Wh)	Discharge Duration (h)	Discharge Power (Watt)	Average Discharge Current (A)	Discharge Capacity (Ah)	Bulk Charge Current (Amp)	Charge Duration (h)
60	6	10	7.5	45.4	25	4.42
80	6	13.3	10.2	61.4	25	6.10
100	6	16.7	12.9	77.8	25	7.69
60	4	15	11.4	45.9	25	4.44
80	4	20	15.6	62.4	25	6.26
100	4	25	19.8	79.4	25	7.96
60	2	30	23.7	47.4	25	4.64
80	2	40	32.5	65	40	6.44
100	2	50	42.05	84.1	40	8.41

## Data Availability

Data is contained within the article.
